# Drift-Free Position Estimation of Periodic or Quasi-Periodic Motion Using Inertial Sensors

**DOI:** 10.3390/s110605931

**Published:** 2011-05-31

**Authors:** Win Tun Latt, Kalyana Chakravarthy Veluvolu, Wei Tech Ang

**Affiliations:** 1 Hamlyn Centre for Robotic Surgery, and Department of Computing, Imperial College London, South Kensington Campus, London SW7 2AZ, UK; E-Mail: w.tun-latt@imperial.ac.uk; 2 School of Electronics Engineering, College of IT Engineering, Kyungpook National University, Daegu 702-701, Korea; 3 School of Mechanical and Aerospace Engineering, Nanyang Technological University, 639798, Singapore; E-Mail: wtang@ntu.edu.sg

**Keywords:** inertial sensors, integration drift, periodic motion, phase-shift, Fourier linear combiner

## Abstract

Position sensing with inertial sensors such as accelerometers and gyroscopes usually requires other aided sensors or prior knowledge of motion characteristics to remove position drift resulting from integration of acceleration or velocity so as to obtain accurate position estimation. A method based on analytical integration has previously been developed to obtain accurate position estimate of periodic or quasi-periodic motion from inertial sensors using prior knowledge of the motion but without using aided sensors. In this paper, a new method is proposed which employs linear filtering stage coupled with adaptive filtering stage to remove drift and attenuation. The prior knowledge of the motion the proposed method requires is only approximate band of frequencies of the motion. Existing adaptive filtering methods based on Fourier series such as weighted-frequency Fourier linear combiner (WFLC), and band-limited multiple Fourier linear combiner (BMFLC) are modified to combine with the proposed method. To validate and compare the performance of the proposed method with the method based on analytical integration, simulation study is performed using periodic signals as well as real physiological tremor data, and real-time experiments are conducted using an ADXL-203 accelerometer. Results demonstrate that the performance of the proposed method outperforms the existing analytical integration method.

## 1. Introduction

It is a well-known fact that the use of numerical integration of acceleration/angular rate information from inertial sensors (accelerometers/gyroscopes) to obtain position/orientation information inherently causes position/orientation errors to grow with time, which is commonly known as “integration drift”. For that reason, estimation of position/orientation using inertial sensors is performed with the help of externally-referenced aided sensors or sensing systems [1,2], or prior knowledge about the motion to correct for the drift.

With the aided sensors or sensing systems, Kalman filters (KF) or extended-Kalman filters (EKF) are commonly used to fuse two sources of information: one coming from the inertial sensors, and the other from aided sensors or sensing systems in an attempt to correct for the drift. For example, correction of orientation drift using EKF and a magnetometer as an aided sensor is described in [3,4]. Correction of position and orientation drift using EKF and ultrasonic sensors as aided sensors is presented in [5]. One of the drawbacks of having to rely on aided sensors to correct for the drift is that the accuracy depends on the update rate, availability, and reliability of the aided sensors.

An example application of the use of inertial sensors with prior knowledge of motion is in human-walking studies. The use of prior knowledge of motion of human walking makes it possible to avoid the use of aided sensors or sensing systems for correction of the drift [6–8], allowing studies of natural walking outside the laboratory. Another application of the use of inertial sensors with prior knowledge of motion is physiological tremor sensing. In physiological tremor sensing for real-time compensation [9,10], zero-phase adaptive filtering algorithms based on truncated Fourier series such as weighted-frequency Fourier linear combiner (WFLC) [11–13] or band-limited multiple Fourier linear combiner (BMFLC) [14–16], which can detect periodic or quasi-periodic signals, are employed.

These algorithms can estimate desired periodic signals from a mixture of desired periodic signals and undesired signals without altering the phase and magnitude of the desired periodic signal. However, the WFLC and the BMFLC have limitations in that the magnitude of the undesired signals comparing to that of the desired periodic signal cannot be too large in order to achieve satisfactory accuracy of the estimate [17,18]. Since the magnitude of the integration drift is too large compared to that of the periodic signal, the algorithms are not well suited for the problem of drift. A method was developed in [19] to obtain the position information from acceleration with analytical integration to avoid drift caused by numerical integration. However, the method in [19] does not consider drift in the obtained acceleration for position estimation and does not compensate for a signal that has already had its phase and magnitude changed by inevitable filters such as a hardware filter which exists at the output of ADXL-203 accelerometers [20].

To obtain drift-free position estimates of periodic or quasi-periodic motion using inertial sensors without employing other aided sensors or sensing systems, one possible solution is to employ linear high-pass filtering of drifted position by choosing a cutoff frequency somewhere between the frequencies of low-frequency drift signal and that of the periodic motion which has relatively high frequency. However, linear filtering inherently introduces phase-shift and attenuation [21], resulting in inaccurate position/orientation estimate.

In this paper, a method is proposed in which a combination of linear filtering and modified-WFLC or modified-BMFLC is employed. The integrated signal will be filtered using a high-pass linear filter. The filtered signal, which is the phase-shifted and attenuated version of the actual desired periodic signal, will then be estimated using WFLC or BMFLC algorithms. Accordingly, the estimate will be the phase-shifted and attenuated version of the actual periodic signal. The estimate of the actual periodic signal is recovered from the phase-shifted and attenuated estimate by compensating for the phase-shift and attenuation introduced by the filter. The compensation is achieved with modification of existing algorithms WFLC and BMFLC.

The main idea behind the proposed method relies on the knowledge of the specification of the linear filter employed in filtering and on that of the frequency content of the desired periodic signal to be estimated. If specification of a filter and frequency of an input signal to the filter are known, the amount of phase-shift and attenuation of the signal at the output of the filter can be known. Using the knowledge of the amount of phase-shift and attenuation introduced by the filter for a particular frequency, compensation for the phase-shift and attenuation of each frequency component in the periodic signal can be performed.

Since WFLC and BMFLC algorithms can provide information on the frequencies in the desired periodic signal together with their respective amplitudes, the algorithms are well suited for the proposed method. In Section 2, the existing algorithms are explained briefly and the modified versions of the algorithms together with the proposed method are presented.

## 2. Methods

In sub-section 2.1 existing methods for zero-phase estimation of periodic signals are discussed first. It should be clear that the existing methods are not the contribution of this paper, but are described briefly to aid readers clearly understand the proposed method which is the contribution of the paper. In sub-section 2.2, the proposed method of drift-free position estimation using inertial sensors is described. Since the proposed method requires the use of an existing estimation method and its modification is required, modification to the existing methods are proposed and described. It should be noted that analytical integration method for drift-free estimation described in [19] does not account for the acceleration drift and the phase-shift and attenuation that has already been introduced by inherent hardware filters. The proposed method can handle these issues very well.

### 2.1. Existing Methods

Weighted Fourier Linear Combiner (WFLC) [11–13] is suitable for estimation of periodic or quasi-periodic motion with single dominant frequency, whereas Bandlimited Multiple-Fourier Linear Combiner (BMFLC) [14–16] is suitable for estimation of band limited signals consisting of multiple frequency components.

#### 2.1.1. Weighted-Frequency Fourier Linear Combiner (WFLC)

**Figure 1.**
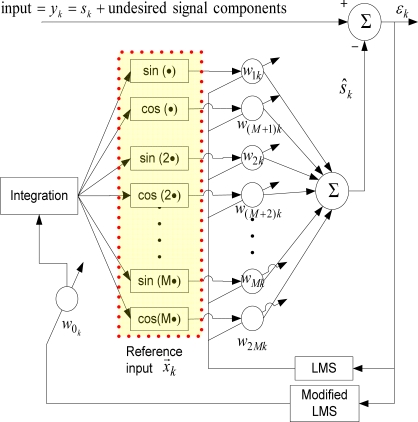
WFLC algorithm.

The WFLC [11–13] algorithm extends the well-known Fourier Linear Combiner (FLC) [22] algorithm to also adapt to the time-varying reference signal frequency, using a modification of the least-mean-square (LMS) algorithm. As FLC only operates at a fixed frequency, the goal of the WFLC algorithm is to adapt to a periodic signal of unknown frequency, phase and amplitude. A block diagram of the WFLC algorithm is shown in Figure 1.

The reference input vector to WFLC, *x⃗*_*k*_ = [*x*_1_*k*__ … *x*_2*M*_*k*__]^*T*^ is:

(1) $$x_{r_{k}}\;=\;\{\begin{array}{l} \text{sin}(rT\;\sum_{t=0}^{k}w_{o_{t}}),\;r\;=\;1,2,\ldots ,\;M\\ \text{cos}\left((r\;-\;M)T\;\sum_{t=0}^{k}w_{o_{t}}\right),\;r\;=\;M\;+\;1,\;M\;+\;2,\ldots ,\;2M \end{array}$$

where *M* is the number of harmonics used, *k* = 1, 2,… represents time-index, *T* is a sampling period. As in FLC, the weight vector is updated using the LMS algorithm:

(2) $${ɛ}_{k}\;=\;y_{k}\;-\;{\vec{w}}_{k}^{T}\cdot {\vec{x}}_{k}$$

(3) $${\vec{w}}_{k+1}\;=\;{\vec{w}}_{k}\;+\;2\mu {\vec{x}}_{k}{ɛ}_{k}$$

where ${\vec{w}}_{k}^{T}\;=\;{\left[w_{1_{k}}\;\cdots w_{2M_{k}}\right]}^{T}$ is the coefficient or weight vector of the reference input, the input to the algorithm *y_k_* contains the desired periodic or quasi-periodic signal which is to be modeled or estimated, *s_k_*, and other undesired components such as noise and low-frequency signals. *μ* is the adaptive gain parameter. The frequency, *w*_0*_k_*_, which is required in the reference input vector, is estimated by modified LMS as follows:

(4) $$w_{0_{k+1}}\;=\;w_{0_{k}}\;+\;2\mu_{0}{ɛ}_{k}\;\sum_{i=1}^{M}i\left(w_{i_{k}}\;x_{M+i_{k}}\;-\;w_{M+i_{k}}\;x_{i_{k}}\right)$$

where *μ*_0_ is adaptive gain parameter.

An estimation of the desired periodic or quasi-periodic signal in the input can be calculated as:

(5) $${\hat{s}}_{k}\;=\;{\vec{w}}_{k}^{T}\cdot {\vec{x}}_{k}$$

However, it should be noted that a good estimation is achieved only when magnitudes of other undesired signal components in *y_k_* are not too large comparing to the magnitude of *s_k_*.

#### 2.1.2. Bandlimited Multiple Fourier Linear Combiner (BMFLC)

**Figure 2.**
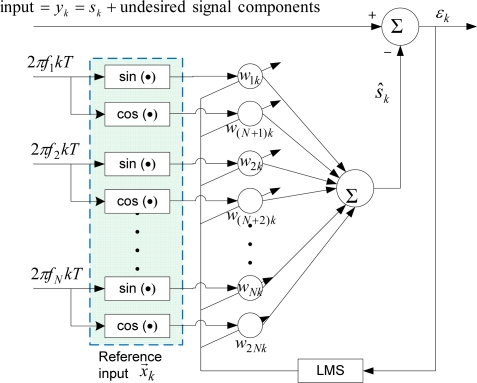
BMFLC algorithm.

One limitation of WFLC is its inability to extract a periodic signal containing more than one dominant frequency. To overcome that, BMFLC [14–16] was developed. This approach relies on choosing a pre-determined band of frequencies based on the prior knowledge of the desired signal’s frequency band. Spacing of frequencies is chosen according to user’s requirement. The block diagram of BMFLC is shown in Figure 2.

The reference input to BMFLC is:

(6) $$x_{r_{k}}\;=\;\{\begin{array}{l} \text{sin}(2\pi f_{r}kT),\;r\;=\;1,2,\ldots ,\;N\\ \text{cos}(2\pi f_{r-N}\;kT),\;r\;=\;N\;+\;1,\;N\;+\;2,\ldots ,\;2N \end{array}$$

where *f_r_* are the frequencies within a given band of interest and *N* represents the number of frequencies used. The frequencies can be an integer as well as a rational number. The weights of BMFLC can be updated via:

(7) $${ɛ}_{k}\;=\;y_{k}\;-\;{\vec{w}}_{k}^{T}\cdot {\vec{x}}_{k}$$

(8) $${\vec{w}}_{k+1}\;=\;{\vec{w}}_{k}\;+\;2\mu {\vec{x}}_{k}{ɛ}_{k}$$

An estimate of the desired signal can be given by:

(9) $${\hat{s}}_{k}\;=\;{\vec{w}}_{k}^{T}\cdot {\vec{x}}_{k}$$

Again, as with WFLC, a good estimation is achieved only when magnitudes of other undesired signal components in *y_k_* are not too large compared to the magnitude of *s_k_*.

### 2.2. Proposed Method of Drift-Free Estimation

**Figure 3.**
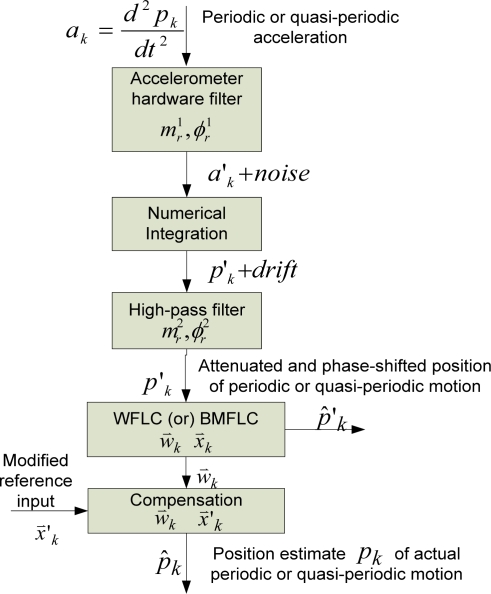
The block diagram showing the steps in estimation of position from accelerometer’s data.

In this section, the proposed method of drift-free estimation of desired periodic or quasi-periodic signal using one of the algorithms described in the previous section, and compensation for the phase-shift and attenuation introduced by the linear filters is described. The proposed method is described using acceleration as representative inertial sensor output. A block diagram describing the method to obtain the position estimate of desired periodic or quasi-periodic motion which is sensed by an accelerometer is shown in Figure 3.

In the figure, *p_k_* is position of periodic or quasi-periodic motion and *a_k_* is corresponding acceleration. The acceleration of the periodic or quasi-periodic motion is attenuated by the inherent hardware filter of the accelerometer. The attenuated acceleration is designated as *a′_k_*. The acceleration is numerically integrated to obtain the position which contains the desired periodic or quasi-periodic motion as well as low-frequency drift. As mentioned in the previous section, if the position obtained from the numerical integration is fed into the input of WFLC or BMFLC algorithm, the estimation performance of the algorithm is severely degraded by the low-frequency drift whose magnitude is too large compared to that of the periodic or quasi-periodic motion signal. Therefore, the low-frequency drift is filtered using a linear high-pass filter. The cutoff frequency and the order of the filter are to be chosen so that the low-frequency drift is removed significantly.

The input signal to WFLC or BMFLC, *p′_k_* is an phase-shifted and attenuated version of the desired periodic or quasi-periodic signal *p_k_* due to filtering by the hardware filter and the high-pass filter. The WFLC or BMFLC operates on the *p′_k_* using a reference input vector [from Equation (1) for WFLC and from Equation (6) for BMFLC] and produces a coefficient or weight vector ${\vec{w}}_{k}^{T}$ and an estimate of *p′_k_*, *p̂′_k_*. Since the goal is to achieve *p_k_*, compensation for the phase-shift and attenuation is to be performed. The compensation is achieved by performing an inner product operation on the weight vector obtained, and the modified reference input vector. The modified reference input vector is obtained by modifying the reference input vector such that the modified reference input vector can compensate for the phase-shift and attenuation. How to obtain the modified reference input vectors in WFLC and BMFLC are described in the later sub-sections.

#### 2.2.1. Modified-WFLC

Although employing a single high-pass filer with an appropriate cutoff frequency might be sufficient to remove the drift before filtering with WFLC or BMFLC algorithms, the modifications made to the algorithms are presented in general for any number of linear filters employed. Therefore, the method can handle inherent filters of the sensors.

Let $m_{r_{k}}^{l}$ and $\varphi_{r_{k}}^{l}$, respectively be the phase-shift and attenuation introduced by *l^th^* filter for *r^th^* harmonic frequency of the fundamental frequency *w*_0*_k_*_ at *k^th^* sample, where *r* = 1, 2,…, *M; l* = 1, 2,…, *P*; and *P* is the number of filters. Then, in WFLC, a modified reference input vector, ${\vec{x}}_{k}^{\prime }\;=\;{\left[x_{1_{k}}^{\prime }\;x_{2_{k}}^{\prime }\;\ldots x_{2M_{k}}^{\prime }\right]}^{T}$ is obtained as follows:

(10) $$x_{r_{k}}^{\prime }\;=\;\{\begin{array}{l} \prod_{l=1}^{P}(1/m_{r_{k}}^{l})\text{sin}(rT\;\sum_{t=0}^{k}w_{o_{t}}\;-\;\sum_{l=1}^{P}\varphi_{r_{k}}^{l}),\;\text{where}\;r\;=\;1,2,\ldots ,\;M\\ \prod_{l=1}^{P}1/m_{r-M_{k}}^{l})\;\text{cos}\left((r\;-\;M)T\;\sum_{t=0}^{k}w_{o_{t}}\;-\;\sum_{l=1}^{P}\varphi_{r-M_{k}}^{l}\right),\;\text{where}\;r\;=\;M\;+\;1,\;M\;+\;2,\ldots ,\;2M \end{array}$$

A compensated estimate (an estimate with compensation for phase-shift and attenuation) of the desired signal becomes:

(11) $${\hat{s}}_{k}\;=\;{\vec{w}}_{k}^{T}\cdot {\vec{x}}_{k}^{\prime }$$

**Figure 4.**
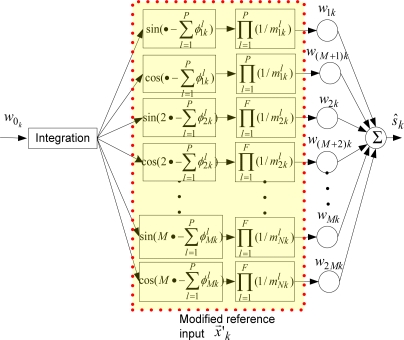
The block diagram of the compensation using the modified reference input in WFLC.

The block diagram of the compensation using the modified reference input in WFLC is shown in Figure 4.

#### 2.2.2. Modified-BMFLC

In the case of BMFLC, assuming $m_{r}^{l}$ and $\varphi_{r}^{l}$, *r* = 1,…,*N; l* = 1,…,*F* respectively are the phase-shift and attenuation introduced by *l^th^* filter for *f_r_* frequency, the modified input vector for compensation is as follows:

(12) $$x_{r_{k}}^{\prime }\;=\;\{\begin{array}{l} \left\{\prod_{l=1}^{F}\frac{1}{m_{r}^{l}}\right\}\;\text{sin}(2\pi f_{r}kT\;-\;\sum_{l=1}^{F}\varphi_{r}^{l}),\;\text{where}\;r\;=\;1,2,\ldots ,\;N\\ \left\{\prod_{l=1}^{F}\frac{1}{m_{r-N}^{l}}\right\}\;\text{cos}(2\pi f_{r-N}kT\;-\;\sum_{l=1}^{F}\varphi_{r-N}^{l}),\;\text{where}\;r\;=\;N\;+\;1,\;N\;+\;2,\ldots 2N \end{array}$$

where *F* is the number of filters. A compensated estimate (an estimate with the compensation) of the desired signal, *ŝ_k_*, can be obtained as in Equation (10):

(13) $${\hat{s}}_{k}\;=\;{\vec{w}}_{k}^{T}\cdot {\vec{x}}_{k}^{\prime }$$

**Figure 5.**
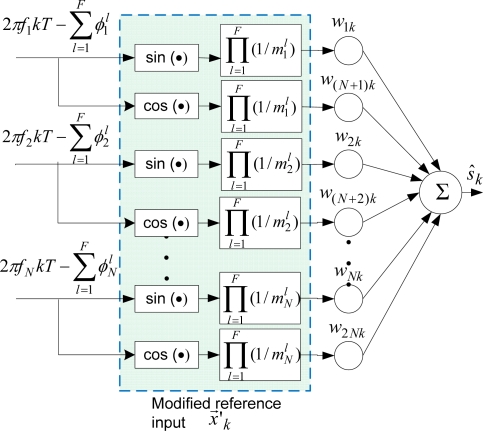
The block diagram of the compensation using the modified reference input in BMFLC.

The block diagram of the compensation using the modified reference input in BMFLC is shown in Figure 5.

## 3. Simulation Methods and Results

In this section, simulations with periodic signals and real physiological tremor data are presented. For illustration of the proposed method in implementation, the simulations are performed with the modified BMFLC algorithm.

### 3.1. Simulation with Periodic Signals

**Figure 6.**
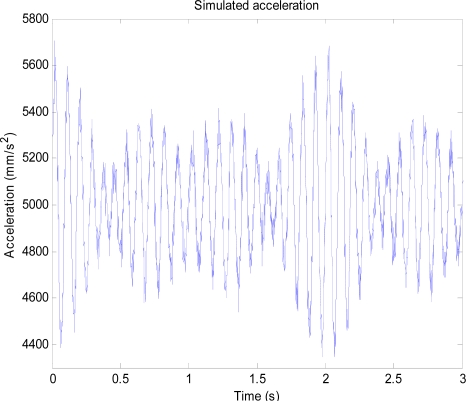
Simulated acceleration output consisting of the sinusoidal acceleration signals with noise and DC offset.

For the simulation with periodic signals, a synthesized periodic acceleration consisting of sinusoidal components with amplitudes 200 mm/s^2^ and frequencies of 10 Hz, 11 Hz, and 11.5 Hz is used as a desired periodic signal whose position is to be estimated. The desired periodic signal is superimposed with Gaussian white noise having a standard deviation of 40 mm/s^2^ and a DC offset value of 5,000 mm/s^2^ to form the simulated noisy acceleration output from an accelerometer. The DC offset is to simulate a sensor bias voltage while noise represents sensor noise. The simulated noisy acceleration output is shown in Figure 6.

**Figure 7.**
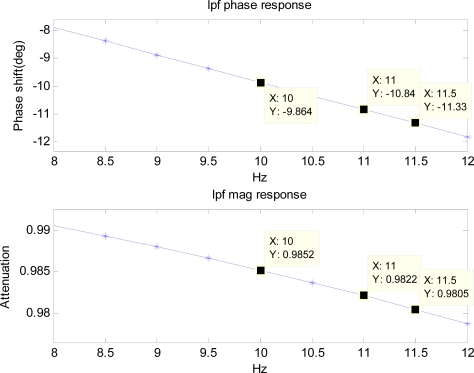
Frequency response of the first-order low-pass filter with a cutoff frequency of 50 Hz.

**Figure 8.**
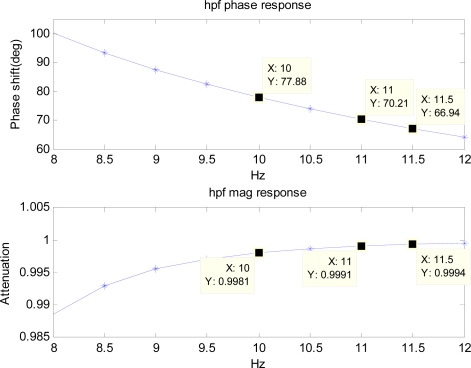
Frequency response of the 4th order high-pass Butterworth filter with a cutoff frequency of 5 Hz.

To simulate the hardware filter of an accelerometer, a first-order low-pass filter with the cutoff frequency of 50 Hz is used. Frequency responses of the low-pass filter and the high-pass filter are pre-calculated and the components in the range of 8 to 12 Hz are shown in Figures 7 and 8.

The cutoff frequency and the order of the high-pass filter are to be chosen such that the filter removes unwanted low-frequency components of drift significantly. Therefore, it depends on the inertial sensor’s noise level that determines the low-frequency components of drift. For noise level used in the simulation, cutoff frequency of 5 Hz, and fourth order are chosen since it removes the unwanted low-frequency drift significantly. The simulated noisy periodic acceleration output is filtered with the low-pass filter before it is numerically double-integrated to obtain the position. The position obtained from the integration is then used with the BMFLC algorithm.

The following parameters are used for implementation of BMFLC: *N* = 5, *μ* = 0.03 and pass-band range for the periodic signal is set from 10 Hz to 12 Hz. The modified reference input for the BMFLC is then:

(14) $$\begin{array}{l} x_{r_{k}}^{\prime }\;=\;\{\begin{array}{l} \left\{\frac{1}{m_{r}^{1}}\;+\;\frac{1}{m_{r}^{2}}\right\}\text{sin}(2\pi f_{r}kT\;-\;\varphi_{r}^{1}\;-\;\varphi_{r}^{2}),\;\text{where}\;r\;=\;1\;\ldots 5;\\ \left\{\frac{1}{m_{r-5}^{1}}\;+\;\frac{1}{m_{r-5}^{2}}\right\}\;\text{cos}(2\pi f_{r-5}kT\;-\;\varphi_{r-5}^{1}\;-\;\varphi_{r-5}^{2}),\;\text{where}\;r\;=\;6\;\ldots \;10; \end{array}\\ \text{with}\;f_{1}\;=\;10,\;f_{2}\;=\;10.5,\;f_{3}\;=\;11,\;f_{4}\;=\;11.5,\;f_{5}\;=\;12 \end{array}$$

The values of $m_{r}^{1}$, $m_{r}^{2}$, $\varphi_{r}^{1}$, and $\varphi_{r}^{2}$ which are known from the frequency response of the low-pass filter and the high-pass filter are induced into the above modified reference input. For example, modified reference input components for 10 Hz frequency *x′*_1*_k_*_ and *x′*_6*_k_*_ are given by:

(15) $$x_{1_{k}}^{\prime }\;=\;\left\{\frac{1}{0.9981}\;+\;\frac{1}{0.9852}\right\}\;\text{sin}(2\pi (10)kT\;-\;77.88\;+\;9.86)$$

(16) $$x_{6_{k}}^{\prime }\;=\;\left\{\frac{1}{0.9981}\;+\;\frac{1}{0.9852}\right\}\;\text{cos}(2\pi (10)kT\;-\;77.88\;+\;9.86)$$

since *m*_1_ = 0.9981, *m*_2_ = 0.9852, *φ*_1_ = 77.88 deg, and *φ*_2_ = −9.86 deg (refer to Figures 7 and 8). Similarly, other components of the modified reference input for other frequencies can be obtained.

To compare estimation performance of the proposed method with that of analytical integration method, the simulated periodic acceleration is filtered by the simulated filter of the accelerometer, and the filtered signal is then double-integrated analytically using the method described in [19]. The same BMFLC parameters are used for the analytical integration method. The unfiltered signal is also double-integrated analytically for comparison between the two methods when the phase-shift and attenuation introduced by the filter of the sensors are negligible.

To show that the proposed method also works very well with gyroscopes, a simulated periodic angular velocity consisting of the same frequency content as the simulated periodic acceleration is used. The amplitude of each component is set at 200 mrad/s. Other settings are kept the same, except that the periodic angular velocity is integrated only once to obtain orientation.

### 3.2. Simulation Results with Periodic Signals

**Figure 9.**
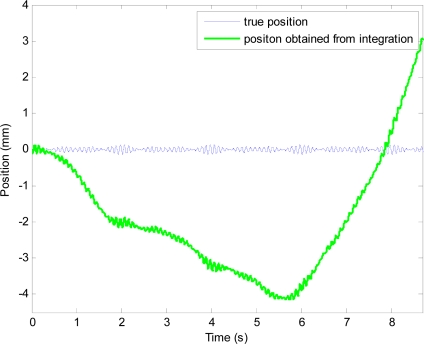
True position and position obtained from double-integration of simulated acceleration.

The position obtained from the integration, which contains periodic position and drift, is shown in Figure 9. After filtering the position obtained from integration with the high-pass filter, drift is removed. However, the filtered position signal is a phase-shifted and attenuated version of the actual periodic motion at 10 Hz.

**Figure 10.**
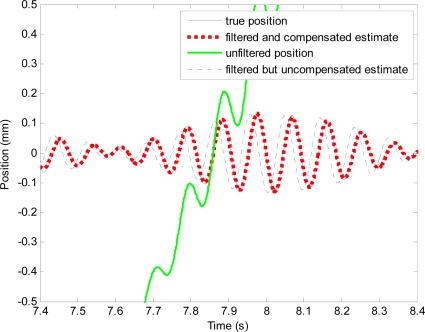
Plots showing effectiveness of the proposed method. The whole plot of the unfiltered position (shown in green line) can be seen in Figure 9.

**Figure 11.**
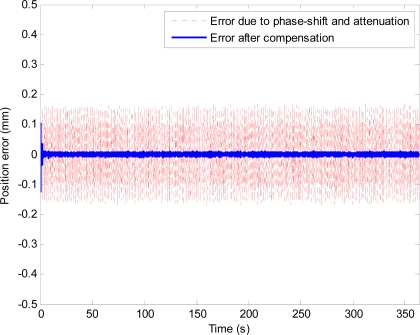
Position estimation error with the high-pass filter and the proposed method.

**Figure 12.**
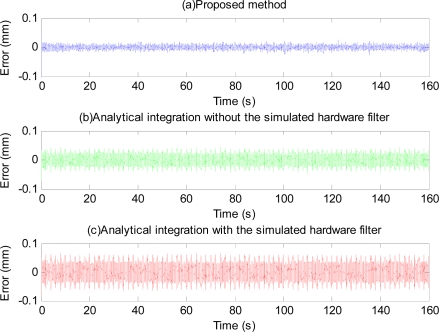
Position estimation error obtained with the proposed method (**a**), the analytical integration without the simulated hardware filter (**b**), and the analytical integration with the filter (**c**).

**Figure 13.**
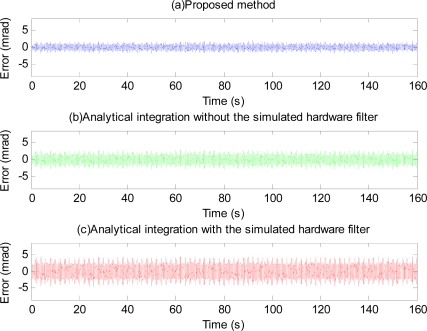
Orientation estimation error obtained with the proposed method (**a**), the analytical integration without the simulated hardware filter (**b**), and the analytical integration with the filter (**c**).

The phase-shift and attenuation is eliminated using the proposed method as can be seen in Figure 10. Position estimation error with the proposed method is shown in Figure 11. Plots and quantitative results of position estimation errors obtained with the proposed method and the analytical integration method are shown in Figure 12 and Table 1, respectively. In the table, the column indicated with “With filter”, and “Without filter” respectively show the results obtained when the simulation is performed with and without the simulated hardware filter. Plots and quantitative results of orientation estimation errors obtained with the proposed method and the analytical integration method are shown in Figure 13 and Table 2, respectively.

### 3.3. Simulation with Real Physiological Tremor Data

In the previous section, simulation of the proposed method using pure periodic signals was presented. In order to show that the method works well with the quasi-periodic signals such as physiological tremor signals since a tremor is approximately a rhythmic (quasi-periodic) signal [23], and can be employed in real-time physiological tremor compensation/cancellation, the method is tested with real physiological tremor data. The tremor data is obtained from surgical instrument tip motion which is measured [24] during micromanipulation tasks performed by subjects using a micro motion sensing system (M2S2) [25]. The instrument tip motion data is filtered off-line using an off-line zero-phase band-pass filter having a pass-band of 5–15 Hz to obtain physiological tremor and remove non-tremulous components such as low-frequency drift, intended motion, and sensor and measurement noise.

To simulate a hardware filter [26] present at the output of ADXL-203 accelerometers employed in tremor compensation instruments [27,28], the tremor data is filtered using a first-order software low-pass filter having a time-constant of 3 ms which is a typical time-constant value of the hardware filter at the output of this type of accelerometer [20]. The filtered tremor data is passed through a BMFLC filter to estimate it. The estimated output of the BMFLC filter has a phase-lag without using the proposed method. The following parameters are used for the implementation of BMFLC: *N* = 21, *μ* = 0.015 and pass-band range for the periodic signal is set from 9 to 11 Hz. The pass-band of 9 to 11 Hz is chosen since most power of tremor frequencies of all the subjects are found from FFT to be within that band.

### 3.4. Simulation Results with Real Physiological Tremor Data

**Figure 14.**
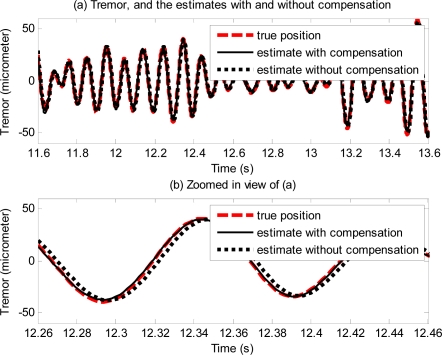
Plots showing the physiological hand tremor (true position) of a subject, estimate of the tremor with and without compensation.

**Figure 15.**
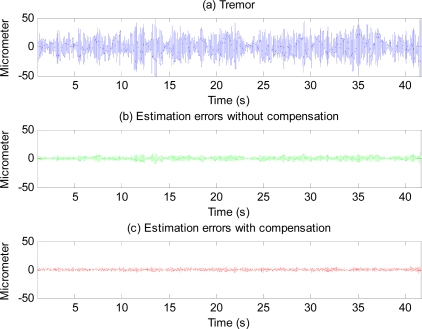
Physiological tremor of a subject (**a**), and estimation errors without compensation for the simulated hardware filter (**b**) and with compensation (**c**).

Figure 14 shows the effectiveness of the proposed method with physiological tremor. A phase-lag can be seen in estimate without compensation. The phase-lag is significantly reduced in the estimate with compensation. Figure 15 shows physiological tremor, and physiological tremor estimation errors with and without compensation. Table 3 shows statistics of root-mean squared (RMS) errors and maximum (peak) errors of ten trials of estimation of different physiological tremor signals with and without compensation, and the percentage of error reduction due to compensation.

## 4. Experiment Methods and Results

**Figure 16.**
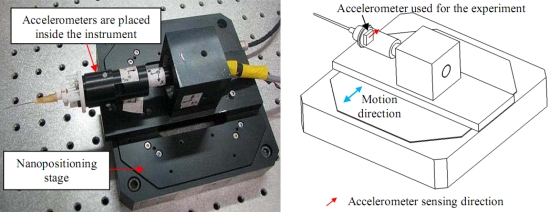
A picture (Left) and a schematic drawing (right) of the experimental setup.

In this section, real-time experiments using an accelerometer as a representative inertial sensor are described. A periodic motion is generated using a commercially available nanopositioning stage (P-561.3CD from Physik Instrumente, Germany) on which a physiological tremor compensation instrument consisting of accelerometers is mounted. The accuracy of the used positioning stage is better than 100 nm. The accelerometers used in the instrument are ADXL-203 accelerometers from Analog Devices. However, only one accelerometer is used for the experiments to show the effectiveness of the proposed method. The setup for the experiment is shown in Figure 16.

The tremor is defined as roughly sinusoidal, and approximately rhythmic [23]. Frequency of physiological tremor lies in the band of 8 to 12 Hz while its amplitude ranges from a few tens to hundreds of microns. To simulate physiological tremor, the nanopositioning stage is programmed to generate 10 Hz sinusoidal motion having peak-to-peak amplitude of 100 μm which is the maximum travel range of the stage. Therefore, peak-to-peak amplitude of the applied acceleration generated by the stage is approximately 400 mm/s^2^ although the accelerometer’s measurement range is ±1.7 g. The voltage output from the accelerometer is acquired at 500 Hz using a 16-bit data acquisition (DAQ) card (PD2-MF-150, United Electronic Industries, Inc, USA).

**Figure 17.**
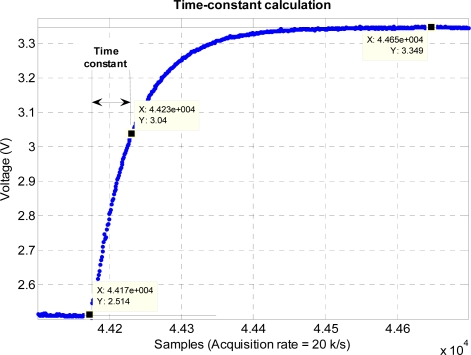
Experimental determination of time-constant of the accelerometer hardware filter.

Before the experiment, static calibration of the accelerometer is performed using a gravity value of 9.81 m/s^2^. The sensitivity value of the accelerometer obtained from the calibration is 980 mV/g. The time constant and hence the specification of the hardware filter of the accelerometer is measured by giving a step input using ST pin of the accelerometer [20] and measuring the time taken for the output to reach 63% of its final value. The sampling rate used in measuring the step response is 20 kHz. The accelerometer hardware filter time constant obtained from the measurement is approximately 3 ms. The step response of the accelerometer is shown in Figure 17. The acquired voltage is then converted to acceleration by using the sensitivity value. The converted acceleration is then double-integrated to obtain position which in turn is filtered using a third-order high-pass filter with the cutoff frequency of 7 Hz. The filtered position is then used with the BMFLC. The parameters used for the implementation of BMFLC are *N* = 7, *μ* = 0.01, and pass-band range for the periodic signal is set from 9 Hz to 12 Hz. Compensation for the phase-shift and attenuation introduced by the hardware filter and the high-pass filter is performed by having dot product between the modified reference input [shown in Equation (12)] and the weight vector produced by the BMFLC.

**Figure 18.**
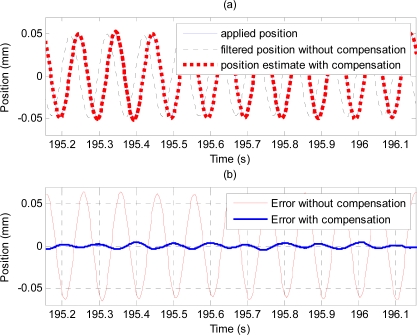
(**a**) Plots of applied position (solid line), position estimate obtained using the proposed compensation method (thick dotted line) and output of the high-pass filter (dashed line), and (**b**) errors with and without compensation.

**Figure 19.**
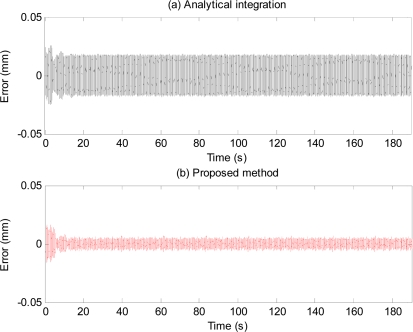
Estimation errors obtained from (**a**) analytical integration of the acceleration, (**b**) the proposed method (*i.e.*, by numerically integrating the acceleration, then high-pass filtering, and compensating for the effects of both hardware filter and HPF filter).

Figure 18 shows plots of applied position (solid line), and position estimate obtained using the proposed method (thick dotted line) and output of the high-pass filter (*i.e.*, without compensation) shown by the dashed line. To compare the performance of the proposed method with that of the analytical integration method, the acceleration signal obtained from the accelerometer is analytically double-integrated using the method described in [19] to obtain the position estimation. Table 4 and Figure 19 show estimation errors with the proposed method and the analytical integration method. To exclude transient errors due to transient adaptation of the algorithm to the signal, calculations of errors are performed from six seconds after the start of the estimation.

## 5. Discussion

As can be seen from Figures 12 and 13, and the quantitative simulation results in Table 1, errors of the proposed method are less than those of the analytical integration method, even in the case that the phase-shift and attenuation introduced by inherent hardware filters are negligible. Higher estimation errors with the analytical integration method are perhaps due to the adaptation of the BMFLC algorithm to the input signal which is noisy. With the proposed method, estimation errors are lesser since the input signal is not noisy due to the linear filtering stage in the proposed method. Errors of the analytical integration method will get larger as the extent of the phase-shift and attenuation introduced by the filters of the inertial sensors are larger while those of the proposed method will remain about the same. Therefore, the proposed method is even more invaluable when the phase-shift and attenuation introduced by the hardware filter are large.

Error plots in Figure 13 and results in Table 2 show the method also works well with gyroscopic data. Plots in Figures 14 and 15, and results shown in Table 3 suggest that the proposed method can track very well physiological tremor signals, which are representative quasi-periodic signals. The phase-shift and attenuation introduced by the simulated hardware filter of an accelerometer are well compensated for and hence accuracy of real-time position estimate is improved by proposed method as can be seen in Figure 14(b). Therefore, the proposed method is useful for real-time physiological tremor compensation [9,10,28]. Experimental results in Table 5 and plots in Figure 18 confirm the effectiveness of the proposed compensation method. As can be seen from the table, the RMS error is reduced by approximately 90% when compensation for the phase-shift and attenuation introduced by both filters is performed. Experiment results in Table 4 and plots in Figure 19 prove superiority of the proposed method over the analytical integration method. The RMS error and maximum error of the proposed method are lesser than that of the analytical integration method by 75% and 52%, respectively.

To achieve a good performance with the proposed method, filter order and cutoff frequency need to be chosen so that the filter removes unwanted low-frequency drift significantly. The choice depends on the sensor noise level and the frequency content of the periodic or quasi-periodic signal. However, a wide range of filter orders and cutoff frequencies are available to be chosen, and hence the choice is not too restrictive. For acceptable reasonable filter order and cutoff frequency, the proposed method outperforms the analytical integration method.

Although the effectiveness of the method is proven using a BMFLC algorithm, it is also valid for the WFLC algorithm. The main reason to employ the WFLC or BMFLC algorithms in the proposed method is because the periodic signals can be modeled by a series of sine and cosine components. The algorithms’ performance is the best for pure periodic motion. If the motion to be estimated is not purely periodic, the algorithms’ performance will be degraded and accuracy of estimation will be affected depending on the degree of non-periodicity.

The performance of the algorithms and hence that of the proposed method also depends on the value of adaptive gain μ. In Figure 19, there exist some errors during a few seconds after the start due to the algorithm’s transient adaptation to the signal. After the algorithm has adapted to the signal, the errors are reduced significantly. The adaptation period can be shortened by increasing the adaptive gain μ at the expense of larger steady-state error. The optimal value of adaptive gain depends on signal-to-noise ratio, and the application requirements. The adaptive gain μ can be chosen to have fast convergence without losing stability. The BMFLC algorithm can be viewed as a series of multiple notch filters, with the width of each notch being directly proportional to μ. The time constant for convergence can be shown to be 1/(2μ) [22]. Typical value of μ is in the range of 0.001 to 0.03 for BMFLC [14–16]. Detailed discussion on the optimal gain can be found in [17,29].

The algorithms (and hence the proposed method) work very well as long as the approximate band of frequencies of the periodic motion is known. If the periodic motion consists of more than one dominant frequency, the BMFLC algorithm should be used. If there is only one dominant frequency with its harmonics in the periodic motion and the dominant frequency is varying slowly, WFLC is a better choice since WFLC can track the periodic motion whose frequency is varying slowly such as that of physiological tremors [17]. Although the method was not tested with motion at very high frequencies (e.g., of the order of kHz) due to the lack of proper equipment (the nanopositioning stage cannot generate very high frequency motion), no compelling reason is seen why the method should not work at these frequencies too.

## Figures and Tables

**Table 1.** Position estimation errors with the proposed method and the analytical integration method with and without the simulated hardware filter.

	**Without filter**		**With filter**	

	**RMS Error**	**Maximum Error**	**RMS Error**	**Maximum Error**

**Error with analytical integration method (μm)**	14.8	43.6	24.1	65.1
**Error with the proposed method (μm)**	6.2	23.1	6.5	23.6
**Error reduction by the proposed method (%)**	58.2	47	73	64
**Error with the proposed method with respect to the true position (%)**	10.5	17.7	11.1	18.1

**Table 2.** Orientation estimation errors with the proposed method and the analytical integration method with and without the simulated hardware filter.

	**Without filter**		**With filter**	

	**RMS Error**	**Maximum Error**	**RMS Error**	**Maximum Error**

**Error with analytical integration method (mrad)**	1.00	2.92	1.64	4.45
**Error with the proposed method (mrad)**	0.54	1.78	0.56	2.03
**Error reduction by the proposed method (%)**	45.77	39.18	66.79	60.1
**Error with the proposed method with respect to the true orientation (%)**	15.01	20.58	15.68	23.49

**Table 3.** Mean and standard deviation of RMS errors and maximum (peak) errors of estimation of physiological tremor signals from ten subjects with and without proposed compensation method.

	**RMS Error**	**Maximum (peak) Error**

**Error without compensation (μm)**	3.46 ± 1.42	15.19 ± 6.85
**Error with compensation (μm)**	2.31 ± 0.73	11.77 ± 7.1
**Error reduction due to compensation (%)**	30.17 ± 11.46	23.73 ± 17.84

**Table 4.** Estimation errors obtained with the analytical integration and the proposed method.

	**RMS Error**	**Maximum Error**

**Analytical integration method (μm)**	11.5	18.9
**The proposed method (μm)**	3	8.9
**Error reduction by the proposed method (%)**	75	52

**Table 5.** RMS error and maximum error of real-time estimation of 10 Hz periodic motion with and without compensation for the phase-shift and attenuation introduced by the filters.

	**RMS Error**	**Maximum Error**

**Error without compensation (μm)**	44	66
**Error with compensation (μm)**	3.0	9
**Error reduction due to compensation (%)**	93	86
